# Quercetin and Isorhamnetin Reduce Benzo[a]pyrene-Induced Genotoxicity by Inducing RAD51 Expression through Downregulation of miR−34a

**DOI:** 10.3390/ijms232113125

**Published:** 2022-10-28

**Authors:** Min Kim, Seung-Cheol Jee, Min-Kyoung Shin, Dong-Hee Han, Kyung-Bin Bu, Seung-Cheol Lee, Bo-Young Jang, Jung-Suk Sung

**Affiliations:** Department of Life Science, Dongguk University-Seoul, Biomedi Campus, 32 Dongguk-ro, Ilsandong-gu, Goyang-si 10326, Gyeonggi-do, Korea

**Keywords:** Benzo[a]pyrene, RAD51, miR−34a, homologous recombination

## Abstract

Benzo[a]pyrene (B[a]P) is metabolized in the liver into highly reactive mutagenic and genotoxic metabolites, which induce carcinogenesis. The mutagenic factors, including B[a]P-7,8-dihydrodiol-9,10-epoxide (BPDE) and reactive oxygen species, generated during B[a]P metabolism can cause DNA damage, such as BPDE-DNA adducts, 8-oxo-dG, and double-strand breaks (DSBs). In this study, we mechanistically investigated the effects of quercetin and its major metabolite isorhamnetin on the repair of B[a]P-induced DNA DSBs. Whole−transcriptome analysis showed that quercetin and isorhamnetin each modulate the expression levels of genes involved in DNA repair, especially those in homologous recombination. RAD51 was identified as a key gene whose expression level was decreased in B[a]P−treated cells and increased by quercetin or isorhamnetin treatment. Furthermore, the number of γH_2_AX foci induced by B[a]P was significantly decreased by quercetin or isorhamnetin, whereas RAD51 mRNA and protein levels were increased. Additionally, among the five microRNAs (miRs) known to downregulate RAD51, miR−34a level was significantly downregulated by quercetin or isorhamnetin. The protective effect of quercetin or isorhamnetin was lower in cells transfected with a miR−34a mimic than in non−transfected cells, and the B[a]P-induced DNA DSBs remained unrepaired. Our results show that quercetin and isorhamnetin each upregulates RAD51 by downregulating miR−34a and thereby suppresses B[a]P-induced DNA damage.

## 1. Introduction

Benzo[a]pyrene (B[a]P), a polycyclic aromatic hydrocarbon, is well-known as a pro-carcinogen classified as a Group 1 carcinogen for humans [1,2,3]. Once B[a]P enters the body, it is metabolized and sequentially oxidized to B[a]P-7,8-epoxide, B[a]P-7,8-dihydrodiol, and B[a]P-7,8-dihydrodiol-9,10-epoxide (BPDE) in the liver. Previous studies have shown that these mutagenic B[a]P-derived metabolites induce DNA DSBs, affecting genomic integrity crucial for cellular functions and causing DNA mutations relevant to cancer development [4,5,6]. BPDE, the main metabolite of B[a]P, intercalates into DNA and covalently binds to nucleophilic guanine bases [7]. Additionally, B[a]P quinone, another B[a]P metabolite, is a highly reactive compound that undergoes redox cycling and thereby generates reactive oxygen species (ROS). Such mutagenic factors cause DNA damage, including double-strand breaks (DSBs) and DNA adducts. Unrepaired DSBs result in chromosomal aberrations, such as gene deletion and chromosome loss, which may ultimately lead to cancers [8]. Therefore, regulation of DNA-repair factors is an important strategy to counter genotoxicity [9]. Numerous studies have shown that BPDE-DNA adducts, the major DNA damage resulting from B[a]P metabolization, can be repaired by cells, especially through the nucleotide excision repair (NER) pathway. DNA DSB is another DNA damage caused by B[a]P. B[a]P-induced DNA DSBs are repaired through a mechanism different from the NER pathway, but little is known about this mechanism.

DSBs are repaired by two mechanisms mainly—non-homologous end-joining and homologous recombination (HR) [10,11]. DSB repair by HR is essential for the recovery of strands of the damaged DNA, contributing to DNA damage tolerance. HR is a DNA repair process that involves invasion of intact DNA by damaged DNA of homologous or similar sequence. Damaged regions are resynthesized using intact DNA as a template [12]. RAD51 is a conserved mediator in HR RAD51 forms a helical nucleoprotein filament on DNA and mediates the homology search and strand-pairing stages of HR [13,14]. MicroRNAs (miRs) can regulate several biological processes, such as cell differentiation, proliferation, and apoptosis, by inhibiting the translation of the complementary target mRNAs [15,16]. A study has shown that miRs regulate approximately 60% of gene transcriptional activity of protein-coding genes [17]. MiR−34a is recognized as a master regulator of tumor suppression through RNA silencing via base-pairing with complementary sequences within mRNA molecules [18].

Quercetin, one of the most well-known flavonoids in plant-based foods, has been studied extensively due to its antioxidant properties [19,20]. Quercetin is methylated at the 3′ position of its B-ring by catechol-O-methyltransferase and thereby converted to isorhamnetin in the liver [21]. Numerous studies have reported that quercetin has a protective effect against B[a]P-induced carcinogenesis [22]. Accordingly, our previous study has suggested that quercetin and isorhamnetin each reduce B[a]P-induced cytotoxicity via modulating drug detoxification metabolism [20]. Whereas most studies have focused on the anti-cancer and antioxidant effects of quercetin against B[a]P-induced cancer development, the effect of quercetin on the repair of B[a]P-induced DNA damage has not been well elucidated [23]. Furthermore, there is no report on the HR mechanisms of quercetin and isorhamnetin against B[a]P-induced genotoxicity. Thus, this study aimed to elucidate the effects of quercetin and its metabolite isorhamnetin on the repair of B[a]P-induced DNA DSBs.

## 2. Results

### 2.1. Quercetin and Isorhamnetin Each Upregulate Homologous Recombination through RAD51 Expression

**Figure 1:**
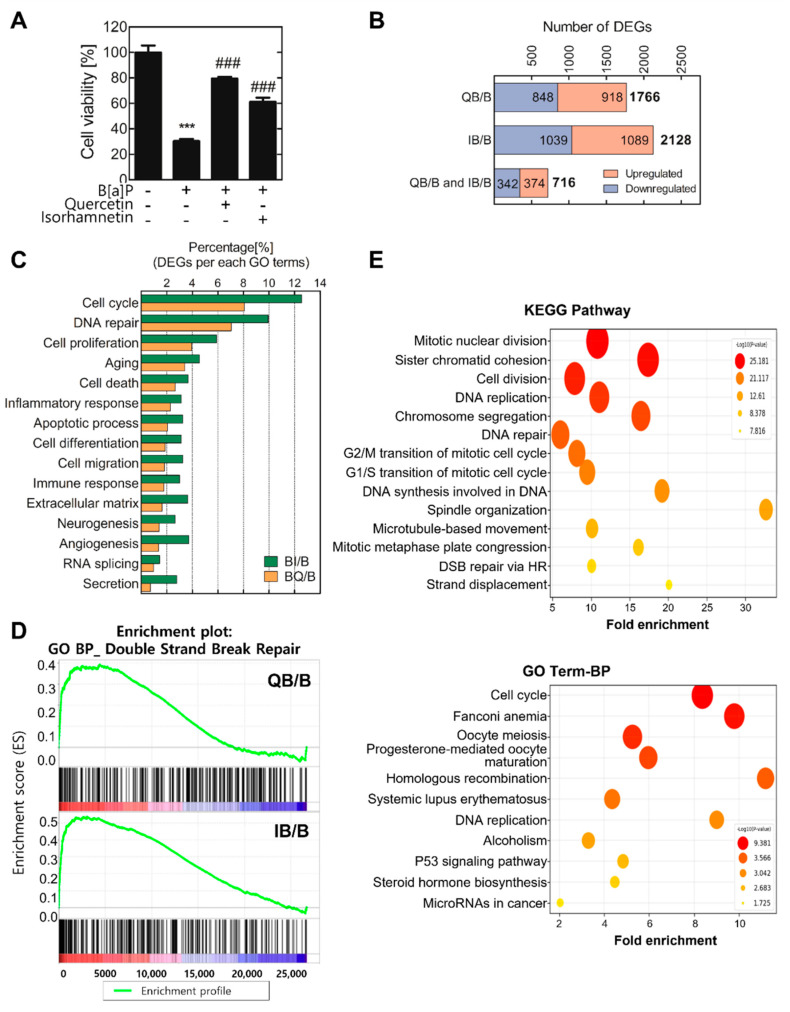
Quercetin and isorhamnetin each modulated expression of genes involved in DNA repair and homologous recombination. (**A**) The viability of cells co−treated with B[a]P and quercetin or isorhamnetin. (**B**) Total cellular RNA was extracted and used for microarray analysis. Differential gene expression analysis was performed by comparing the mRNA levels in B[a]P−treated cells with the levels in cells co−treated with B[a]P−treated and quercetin or isorhamnetin. (**C**) Gene ontology (GO) analysis was conducted using the 716 differentially expressed genes (DEGs, ≥1.5 folds). (**D**) Enrichment analysis was conducted on the DEGs (≥1.5 folds). The significantly affected pathways or GO terms are listed along the y-axes. The x-axes represent fold enrichment. The pathways or terms with a *p*-value < 0.05 were considered significantly enriched. (**E**) Gene set enrichment analysis plot shows that the DEGs are significantly enriched in DNA double-strand break repair. *** *p* < 0.001 compared with the control; ### *p* < 0.001 compared with B[a]P−treated cells. Q; quercetin, I; isorhamnetin, B; B[a]P.

In a previous study, we confirmed that quercetin and isorhamnetin have protective and detoxification effects against B[a]P-induced toxicity [20]. To validate the cytotoxicity of B[a]P and the protective effects of quercetin and its metabolite isorhamnetin, the cell viability was evaluated after 10 μM of B[a]P treatment with or without quercetin or isorhamnetin (Figure 1A). The results showed that B[a]P reduced cell viability to 30% of control, and quercetin and isorhamnetin mitigated the B[a]P-induced cytotoxicity. The most effective concentrations of quercetin and isorhamnetin were both 5 μM, and thus the following experiments were performed at this concentration of quercetin or isorhamnetin. To identify the DEGs between B[a]P−treated cells and those co−treated with B[a]P and quercetin or isorhamnetin, total RNA was isolated from the treated cells and subjected to microarray analysis. Among the 44,629 RNAs represented on the arrays, 1766 and 2128 RNAs were differentially expressed at least 1.5 folds (FC ≥ 1.5 or <0.67, normalized log_2_ > 3). Especially, the expression levels of 716 DEGs were commonly modulated by quercetin and isorhamnetin co−treatments with B[a]P (Figure 1B). Then, the 716 DEGs were analyzed for gene ontology analysis to determine the functional groups in which they participate. The results showed that the DEGs are mainly involved in the cell cycle, DNA repair, and cell proliferation (Figure 1C). Additionally, gene ontology enrichment analyses using the KEGG and DAVID databases were conducted to identify the enriched biological processes among the 716 DEGs (Figure 1D). The results showed that these DEGs were involved in cell division, DNA replication, DNA repair, cell cycle, DNA synthesis involved in DNA repair, DNA DSB repair via HR, and microRNAs in cancer. Among these processes, we focused on DNA DSB repair via HR. GSEA analysis of the commonly modulated 716 DEGs showed that quercetin or isorhamnetin co−treatment with B[a]P tends to upregulate the RNA levels of genes involved in DSB repair, compared with the levels in cells treated only with B[a]P (Figure 1E).

**Figure 2:**
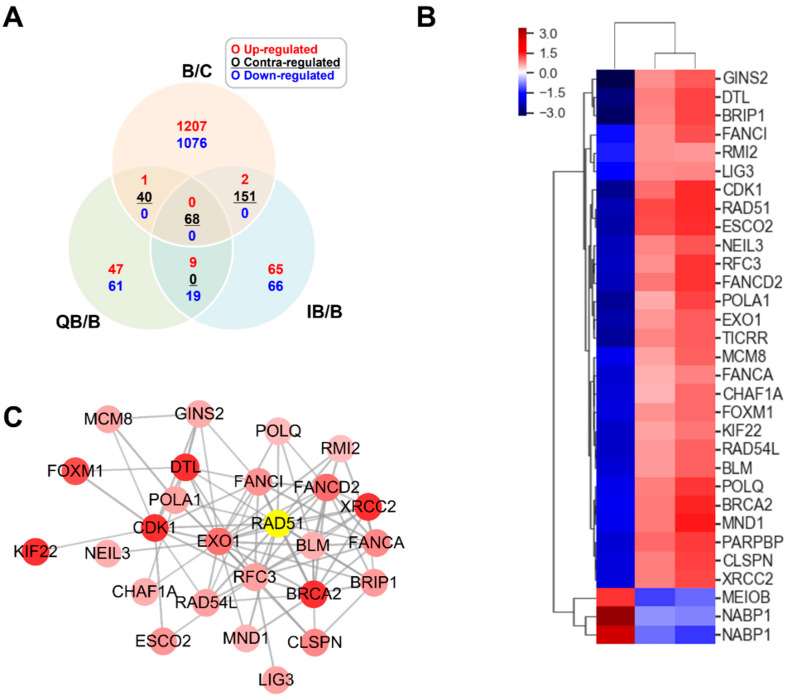
Quercetin or isorhamnetin suppressed the B[a]P-induced downregulation of RAD51. (**A**) Among the differentially expressed genes (DEGs, ≥2.0 folds, presented on a Venn diagram), between B[a]P−treated cells and cells co−treated with B[a]P and quercetin or isorhamnetin, 68 genes are contra-regulated by quercetin and isorhamnetin each. (**B**) Among the 68 DEGs, 31 genes are related to DNA repair. The heatmap of hierarchical clustering of the 31 genes was constructed using their z-scores. Red and blue indicate upregulation and downregulation, respectively. (**C**) The 31 DEGs were subjected to protein–protein interaction (PPI) analysis according to the STRING database. The module enrichment was based on the PPI network with a cutoff criterion of MCODE score > 0.8. Each node and line represent a gene and co-expression relationship, respectively. The bold lines indicate that the connected nodes have a strong co-expression relationship. GO; gene ontology, Q; quercetin, I; isorhamnetin, B; B[a]P.

The gene expression levels were increased or decreased by B[a]P, and these altered expression levels returned to the baseline by co−treatment with quercetin or isorhamnetin. Those results were displayed on a Venn diagram. (Figure 2A) and the results show that there are the most significant 68 DEGs (FC ≥ 2.0 or <0.5, normalized log_2_ > 3). Among these top 68 DEGs, we focused on the 31 DEGs involved in DNA-repair mechanisms. To analyze the similarity among these 31 DEGs, they were standardized to the z-score and then subjected to hierarchical clustering. To calculate the z-scores, each raw data of RNA level was normalized using a z−transformation. The results showed that the genes involved in DNA repair tend to be downregulated by B[a]P and upregulated by quercetin or isorhamnetin.

PPI networks provide comprehensive overviews of sub-cellular interactions [24]. Therefore, by using the Cytoscape program according to the STRING database, we constructed a PPI network of the 31 DEGs involved in DNA-repair mechanisms. The results from the PPI network analysis showed that, among these DEGs, 26 are associated with each other (Figure 2C). Especially, *RAD51* is located at the center of the network, and it is the most significantly upregulated gene by quercetin or isorhamnetin (Table 1).

### 2.2. Quercetin and Isorhamnetin Reduce DNA DSBs and Increase RAD51 Expression

**Figure 3:**
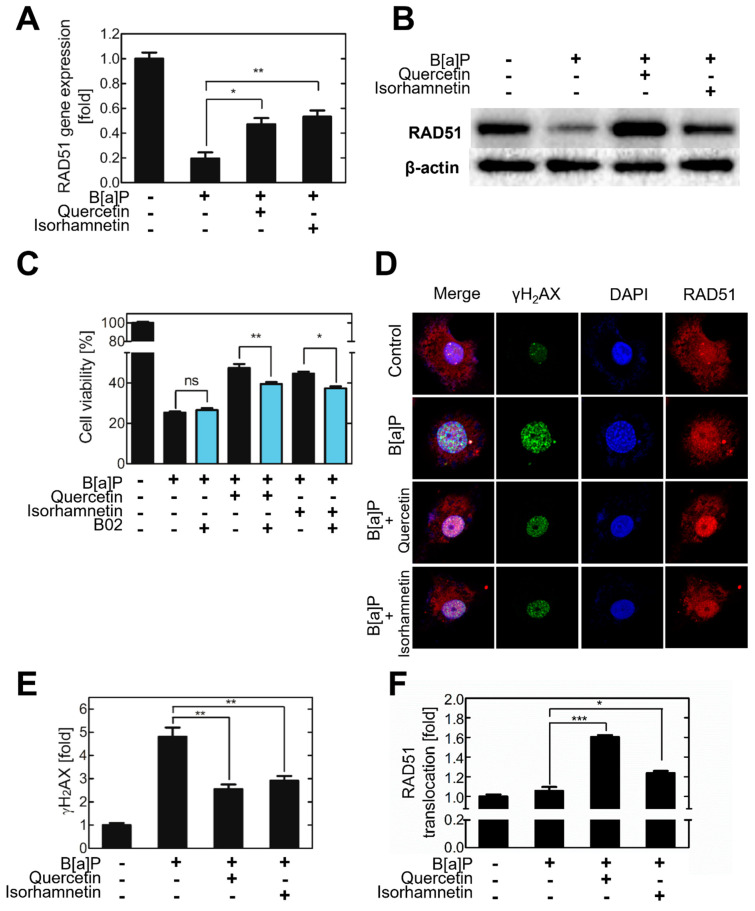
Quercetin or isorhamnetin upregulated RAD51 and thereby decreased the number of DSBs. (**A**) To validate the expression level of RAD51 from the microarray analysis results, cellular *RAD51* mRNA was quantitated via reverse transcription−quantitative polymerase chain reaction. (**B**) The protein level of RAD51 was evaluated via Western blotting. (**C**) The viability of cells co−treated with quercetin or isorhamnetin and B[a]P after administration of a RAD51 inhibitor was evaluated. (**D**) γH_2_AX and RAD51 expression patterns were evaluated via fluorescence microscopy by using Alexa 488 and 555, respectively. The fluorescence intensity from (**E**) γH_2_AX is expressed as a bar graph. (**F**) The translocation of RAD51 to the nucleus was quantified and expressed as a bar graph. ns; not significant, * *p* < 0.05, ** *p* < 0.01, *** *p* < 0.001, C; control, Q; quercetin, I; isorhamnetin, B; B[a]P, B02; inhibitor of RAD51.

To validate whether the RAD51 is modulated by B[a]P, quercetin, and isorhamnetin, *RAD51* mRNA levels were analyzed in treated cells. The results showed that B[a]P reduced the *RAD51* mRNA level in HepG2 cells, whereas quercetin or isorhamnetin increased it (Figure 3A). To confirm that *RAD51* expression is modulated by quercetin and isorhamnetin, the RAD51 inhibitor B02 was administered to HepG2 cells co−treated with quercetin or isorhamnetin and B[a]P. The results showed that the Rad51 inhibitor B02 significantly suppressed the quercetin- or isorhamnetin-induced recovery in cell viability (Figure 3C). To further investigate the effects of quercetin and isorhamnetin on DNA DSBs and RAD51 expression, the γH_2_AX and RAD51 protein levels in HepG2 cells were evaluated via immunofluorescence analysis. γH_2_AX intensity was found to be higher in the B[a]P−treated cells than in the untreated control cells. However, quercetin or isorhamnetin co−treatment suppressed the B[a]P-induced increase in γH_2_AX intensity (Figure 3D,E). Similarly, RAD51 fluorescence intensity was decreased in B[a]P−treated cells relative to the level in the control but was at the baseline level in B[a]P−treated cells co−treated with quercetin or isorhamnetin (Figure 3C). Notably, in B[a]P−treated cells, RAD51 was localized more in the nucleus than in the cytoplasm, indicating that B[a]P induces translocation of RAD51 from the cytoplasm to the nucleus.

### 2.3. Quercetin and Isorhamnetin Decrease Cellular miR−34a Levels Which Increased by B[a]P

**Figure 4:**
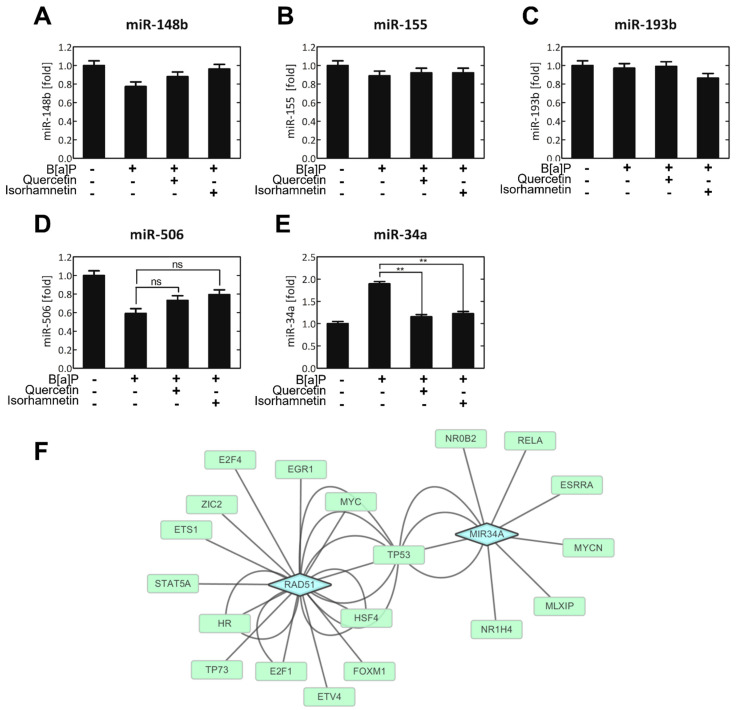
Quercetin or isorhamnetin downregulated the cellular level of miR−34a, which targets the *RAD51* transcript. The cellular levels of (**A**) miR−148b, (**B**) miR−155, (**C**) miR−193b, (**D**) miR−506, and (**E**) miR−34a were assessed. (**F**) Pathway analysis based on Signaling Pathways Integrated Knowledge Engine (Spike) database was performed to confirm the relation between RAD51 and miR−34a. ns; not significant. ** *p* < 0.01, C; control, Q; quercetin, I; isorhamnetin, B; B[a]P.

Intracellular levels of five miRs known to regulate RAD51 were analyzed. Quercetin and isorhamnetin each significantly downregulated miR−34a but no other miRs, including miR148b, miR155, miR193b, and miR506 (Figure 4A–E). B[a]P, quercetin, and isorhamnetin did not affect the cellular level of miR−155, miR−148b, or miR−193b (Figure 4A–C). B[a]P significantly decreased cellular miR−506 level, but quercetin or isorhamnetin co−treatment had no significant effect on the B[a]P-induced decrease in miR−506 level (Figure 4D). B[a]P increased the miR−34a level to 2−fold of the control level, and quercetin or isorhamnetin suppressed this effect (Figure 4E). Subsequently, network analysis was conducted to confirm the relationship between RAD51 and miR−34a (Figure 4F).

### 2.4. Transfection of the Cells with a miR−34a Mimic Mitigate the Effects of Quercetin and Isorhamnetin on DSBs and RAD51 Expression

**Figure 5:**
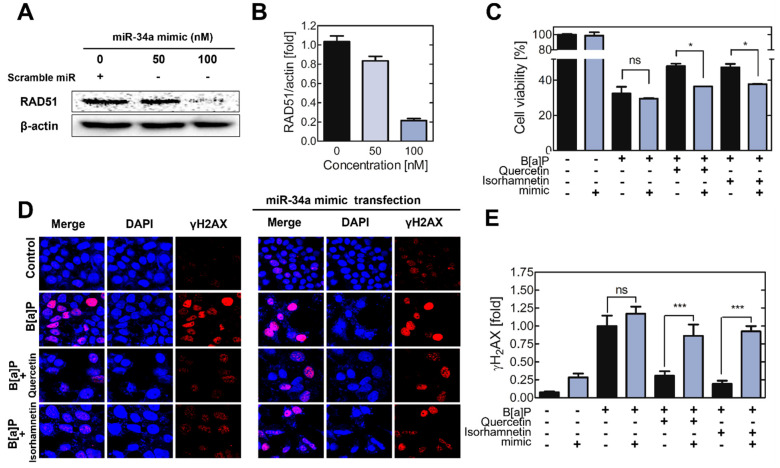
The effects of quercetin and isorhamnetin on cells transfected with a miR−34a mimic. (**A**) The protein level of RAD51 in cells transfected with a miR−34a mimic was assessed. (**B**) The relative RAD51 levels in variously treated cells are expressed as a bar graph. β−actin was used as an internal control in (**A**,**B**). (**C**) The viability of the cells transfected with the miR−34a mimic was analyzed after they were co−treated with B[a]P and quercetin or isorhamnetin. (**D**) The expression pattern of γH_2_AX in the cells transfected with the miR−34a mimic was compared with that in non−transfected cells. (**E**) The fluorescence intensity from γH_2_AX was calculated and expressed as a bar graph. * *p* < 0.05, *** *p* < 0.001, ns; not significant, C; control, Q; quercetin, I; isorhamnetin, B; B[a]P, m; mimic transfection.

To validate the effect of miR34a on RAD51 expression and cell viability, cells were transfected with a miR−34a mimic, their viability was analyzed after B[a]P co−treatment with quercetin or isorhamnetin. First, the RAD51 protein level was decreased after transfection of the cells with the miR−34a mimic (Figure 5A,B). In the cells transfected with the mimic, the protective effects of quercetin and isorhamnetin were reduced compared with the effects in non−transfected cells (Figure 5C). The results of the γH2AX assay showed that administration of the miR−34a mimic suppressed the effects of quercetin and isorhamnetin on the B[a]P-induced DNA DSBs (Figure 5D,E).

## 3. Discussion

B[a]P, one of the carcinogen, is ubiquitous compound found in cigarette smoke, burning coal smoke, and various foods, especially fire-grilled red meat [25]. Once B[a]P is absorbed into the human body, it is metabolized in the liver and transformed into various metabolites. Previous studies have shown that mutagenic factors, such as ROS and BPDE, generated during B[a]P metabolism, induce DNA DSBs, affecting genomic integrity, which is crucial for cellular functions [5,6]. Additionally, a study has shown that unrepaired DNA lesions inhibit transcription and replication, leading to mutations and cancer [26]. Therefore, regulation of DNA-repair factors is an important strategy to counter genotoxicity [9]. Since BPDE is the main metabolite of B[a]P, studies on B[a]P-induced DNA damage and the ensuing DNA-repair mechanism have been mostly concerned with BPDE-DNA adducts and their repair mechanism, the NER pathway [27,28,29]. HepG2 cells are a type of liver cell used in drug metabolism studies due to their property of drug-induced metabolic enzyme expression. In addition, it is also reported that xenobiotics including PAHs significantly induced CYP1A1 and CYP1B1 expression and are metabolized in HepG2 cells [30].

Whereas most studies have focused on the anti-cancer and antioxidant effects of quercetin against B[a]P−induced cancer development, the effect of quercetin on the repair of B[a]P-induced DNA damage has not been well elucidated. In addition, although the plasma isorhamnetin level after quercetin ingestion is approximately five times more than the plasma quercetin level [31], isorhamnetin has not been as well-studied as quercetin, and B[a]P-related isorhamnetin studies are lacking. Accordingly, this study provides new mechanistic insights into the effects of quercetin and isorhamnetin on the repair of B[a]P-induced DNA DSBs.

Transcriptomics analyses can provide comprehensive information about expression levels of a vast number of genes, whereby molecular mechanisms underlying complex biological processes can be elucidated [32,33]. Herein, to discover the significant genes, including miRs, related to quercetin– or isorhamnetin–induced attenuation of B[a]P-induced DNA damage, microarrays and bioinformatics analyses were conducted. The common DEGs were found to be mainly involved in the cell cycle, DNA repair, and cell proliferation. Likewise, the enriched GO biological processes were cell cycle, homologous recombination, DNA replication, and microRNAs in cancer, all of which are processes associated with DNA DSB repair (Figure 1C–E). Generally, upon sensing DNA damage, the cell cycle is arrested, followed by activation of DNA-repair mechanisms [34]. Cell-cycle arrest affects the rate of cell proliferation [35], and thus the cell cycle, DNA repair, and cell proliferation are highly associated with each other. Our results suggest that quercetin and isorhamnetin each modulate biological processes related to DNA repair, including DNA synthesis and HR, thereby affecting the cell cycle. Additionally, considering that microRNAs significantly modulated, we hypothesized that these miRNAs were related to a process in DNA repair, HR.

DNA DSB repair by HR involves the invasion of the broken DNA strands into a homologous DNA sequence, which is then used as a replication template [36,37]. This process requires RAD51, which forms a helical nucleoprotein filament on DNA and mediates the homology search and strand-pairing during HR [14]. RAD51 is very frequently overexpressed in cancer and is considered a key protein that plays an important role in cancer cell development and survival. Therefore, direct targeting of RAD51 to reduce its activity and expression is one strategy to sensitize and overcome cancer cells resistant to conventional DNA damage therapy [38]. The Rad51 inhibitor significantly suppressed the quercetin− or isorhamnetin−induced recovery of the viability of B[a]P−treated cells (Figure 3C). This result indicates that RAD51 is an important factor underlying the cytoprotective effects of quercetin and isorhamnetin. The phosphorylated histone variant H_2_AX, which forms γH_2_AX, is an early cellular response induced by a DNA DSB and is therefore commonly used for quantification of DNA damage, especially DSBs. [39]. In our results, the B[a]P−treated cells had higher intensity of γH_2_AX than the control cells, indicating that B[a]P induces DSBs on the genomic DNA. However, quercetin or isorhamnetin co−treatment reduced the intensity of γH_2_AX in B[a]P−treated cells, indicating that quercetin and isorhamnetin each suppresses B[a]P-induced DSB (Figure 3D,E).

It was reported that DNA damage induces cytoplasmic Rad51 transport to the nucleus [40]. It is noteworthy that, in the absence of the DNA-damage inducer B[a]P, RAD51 was evenly distributed between the cytoplasm and nucleus. However, in the B[a]P−treated cells, it was found concentrated in the nucleus rather than in the cytoplasm, indicating that B[a]P induces translocation of RAD51 from the cytoplasm to the nucleus. Moreover, our results showed that quercetin or isorhamnetin even further increased the nuclear translocation of RAD51 in B[a]P−treated cells.

The changes in RNA levels we observed indicate that the transcription of the related genes was modulated. miRs regulate approximately 60% of the transcripts of protein-coding genes [17]. It has also been reported that some miRs are regulated in response to DNA damage. miRNAs, including miR−24, 138, 96 and 182, have been involved in DNA damage response, DNA repair and cellular sensitivity to DNA damaging agents [41]. To date, at least five miRs have been identified to downregulate RAD51—miR−155, miR−148b, miR−193b, miR−506, and miR−34a [42,43,44,45]. Therefore, we analyzed the cellular levels of these five miRs in treated cells and observed that quercetin and isorhamnetin each significantly downregulated miR−34a but no other miRs (Figure 4A–E). Then, the relationship between RAD51 and miR−34a was confirmed via network analysis (Figure 4F). This analysis revealed that miR−34a modulates the level of TP53, which is a transcription factor regulating the expression of the *RAD51* gene [46]. TP53 plays crucial roles in the control of the cell cycle and tumor suppressor [47]. Recent studies have reported TP53-binding sites in the promoter of the *RAD51* gene [48].

**Figure 6:**
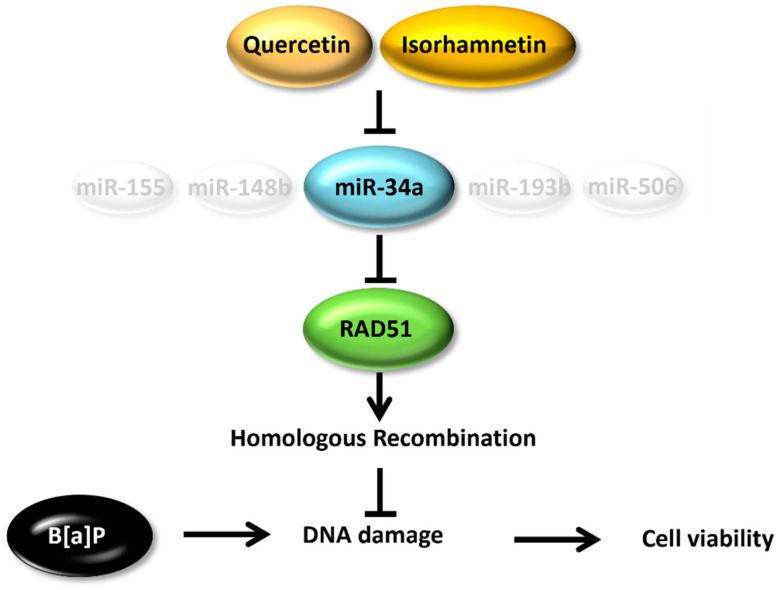
The mechanistic model of quercetin and isorhamnetin against B[a]P-induced genotoxicity.

MiR−34a is recognized as a master regulator of tumor suppression through RNA silencing [18]. Attenuation of RAD51 expression is generally associated with decreased HR and increased sensitivity of cells to DNA damaging agents [49]. Our results showed that the RAD51 protein level was decreased after administration of the miR−34a mimic (Figure 5A,B). The viability of cells transfected with 200 nM mimic was analyzed after their B[a]P co−treatment with quercetin or isorhamnetin. We hypothesized that if indeed quercetin and isorhamnetin each upregulated RAD51 by downregulating miR−34a, their effects would be reduced by increasing miR−34a activity through the administration of the mimic. The protective effects of quercetin and isorhamnetin on cell viability were reduced in the cells transfected with the miR−34a mimic compared with the viability of the non−transfected cells (Figure 5C). In addition, the miR−34a mimic transfection mitigates the effect of quercetin and isorhamnetin on genetic integrity (Figure 5D,E). Recent studies reported that translational regulation of RAD51 modulates HR. According to Enjie Li et al. METTL3 upregulates the expression of RAD51, resulting in enhanced DNA repair by homologous recombination [50]. In addition, a study shows that Elp1 regulates RAD51-mediated HR via translational regulation of RAD51 [51]. Our results show that miR34a, which regulated the RAD51 level, is significantly induced by B[a]P and returned to normal state by quercetin and isorhamnetin. Besides, it was confirmed that both mRNA and protein level of RAD51 were decreased by B[a]P, and restored by quercetin and isorhamnetin. Taken together, it is suggested that quercetin and isorhamnetin could modulate the HR by miR34a/RAD51 axis (Figure 6).

## 4. Materials and Methods

### 4.1. Reagents

Quercetin, isorhamnetin, B[a]P, 4,6-diamidino-2-phenylindole dihydrochloride (DAPI), RAD51 inhibitor B02, dimethyl sulfoxide, and Triton X-100 were purchased from Sigma-Aldrich (St. Louis, MO, USA). The RAD51 antibody was purchased from abcam (ab63801, Cambridge, UK), and γH_2_AX antibody purchased from Millipore (05-636, Burlington, MA, USA). Anti-mouse Alexa 488 (4408S) and Anti rabbit IgG-HRP (7074S) was purchased from cell signaling technology (Danvers, MA, USA). The antibodies of goat anti-rabbit IgG−tRICT (sc-3841), beta-actin (sc 47778), and m-IgGκ BP-HRP (sc-516102) were purchased from Santa Cruz biotechnology (Dallas, TX, USA)

### 4.2. Cell Culture and Treatment

The HepG2 cell line was purchased from the American Type Culture Collection (Manassas, VA, USA). HepG2 cells were cultured in a 150 mm^2^ cell-culture dish with minimum essential medium (MEM; Welgene, Daegu, Republic of Korea) containing 10% FBS (Gibco, Grand Island, NY, USA), 100 U/mL penicillin and streptomycin (Welgene, Daegu, Republic of Korea), and 1 mM sodium pyruvate (Welgene, Daegu, Republic of Korea). All cells were incubated at 37 ℃ with 5% CO_2_, and the medium was changed every other day. For the experiments, all cells were seeded at the density of 3 × 10^4^ cells/cm^2^ and cultured in the above medium with various concentrations of B[a]P with or without quercetin or isorhamnetin for 48 h. Afterward, the cells were lysed or used for further analysis.

### 4.3. Cell-Viability Analysis

Cell viability was assessed using the EZ-CYTOX reagent (DOGEN, Seoul, Republic of Korea). Cultured cells were transferred into 96-well plates (1 × 10^4^ cells/well). After treatment of quercetin or isorhamnetin with B[a]P on HepG2 for 24 h and 48 h, the culture medium was replaced with the medium containing the EZ-CYTOX reagent. After 2 h of incubation, the absorbance at 450 nm was measured using a microplate reader (Molecular Devices, San Jose, CA, USA). The cell viability in each chemical−treated sample was expressed as a relative value to that in the vehicle−treated control group.

### 4.4. mRNA Extraction

Total RNA was extracted using TRIzol reagent (Life Technologies, Carlsbad, CA, USA) by following the manufacturer’s protocol. The extracted RNA samples were dissolved in H_2_O. To verify the integrity of the samples, they were mixed with the 6× gel-loading dye containing formamide and electrophoresed on a 1% agarose gel (Sigma-Aldrich, St. Louis, MA, USA) with ethidium bromide (Sigma-Aldrich, St. Louis, MA, USA) in Tris-acetic acid-EDTA buffer (Biosesang, Gyeonggi, Republic of Korea) for 40 min at 50 V. RNA quantity and quality were assessed using a Nanodrop-2000 (Thermo Fisher Scientific, Waltham, MA, USA), and the 260/280 nm absorbance ratio of all the samples were >1.8. These RNA samples were used for microarray or reverse transcription-quantitative polymerase chain reaction (RT-qPCR). Only high-quality RNA samples (RNA integrity number > 7.0) were used in the experiments.

### 4.5. Microarray Analysis

To analyze the global gene-expression profiles, we had ebiogen Inc. (Seoul, Republic of Korea) perform microarray analyses using Affymetrix GeneChips^TM^ (Thermofisher, Waltham, MA, USA) and process the raw data. Briefly, total RNA was reverse−transcribed into double-stranded cDNA. The fragmented cDNA was hybridized to the Affymetrix arrays for 16 h at 45 °C by following the instructions in the manual of GeneChip Whole Transcript (WT) Sense Target Labeling Assay (Affymetrix, Santa Clara, CA, USA). Afterward, the arrays were stained using Streptavidin Phycoerythrin, washed in a GeneChip Fluidics Station 450, and scanned using a GeneChip Array Scanner 3000 7G. The arrays were scanned using an Affymetrix Model 3000 G7 scanner, and the image data were extracted using the Affymetrix Command Console software (version 1.1). The raw expression data were generated using Transcriptome Analysis Console 4.0.1 (Affymetrix, Santa Clara, CA, USA). Data mining and graphic visualization were performed using ExDEGA (Ebiogen Inc., Seoul, Republic of Korea).

### 4.6. Gene Ontology and Pathway Analysis

Differentially expressed genes (DEGs) between samples were subjected to gene ontology and KEGG pathway analyses. To minimize false counts, the DEGs between B[a]P−treated samples and B[a]P−treated samples co−treated with quercetin or isorhamnetin were sorted in the condition of fold change (FC) > 2.0 or <0.50 and log_2_-normalized read count > 4.0. Gene classification was based on searches performed using the DAVID (Accessed 2 February 2022, https://david.ncifcrf.gov/) and Medline databases (Accessed 2 February 2022, http://www.ncbi.nlm.nih.gov/).

### 4.7. Protein–Protein Interaction (PPI) Mapping

To assess a functional interaction network, PPI mapping of the sorted DEGs was performed based on the global gene-expression profiles. The interactions of each protein were predicted using the human interactome in the STRING database (version 11.5; Accessed on 2 February 2022, http://string-db.org/). The STRING database is based on a combination of various sources, such as experimental verification, text-mining, and co-expression.

### 4.8. Gene Set Enrichment Analysis (GSEA)

GSEA (Accessed on 2 February 2022, https://www.gsea-msigdb.org/) was performed to compare the normalized read counts of all the genes as raw microarray data. GSEA calculates the enrichment score (ES) and the normalized ES (NES), which indicate whether the expression level of a gene is over-represented among the DEGs [52].

### 4.9. RT-qPCR

cDNA was transcribed from 2000 ng total RNA by using M-MLV (Moloney Murine Leukemia Virus) Reverse Transcriptase (ELPIS-BIOTECH, Daejeon, Republic of Korea) and used for qPCR (CFX Connect™ Real−time PCR Detection System; Bio-Rad, CA, USA). Amplicons were quantitated in real−time by using SYBR Green PCR Master Mix (KAPA, MA, USA). PCR was performed using the following cycling conditions: hot-start initial denaturation at 95 °C for 3 min, 50 cycles of denaturation at 95 °C for 10 s, annealing at 60 °C for 10 s, and extension at 72 °C for 10 s.

### 4.10. Immunofluorescence Analysis

Compound−treated cells were fixed with 4% formaldehyde (Sigma-Aldrich, St. Louis, MA, USA) for 15 min and then incubated with 0.25% Triton X-100. Afterward, the samples were blocked with 1% bovine serum albumin (Sigma-Aldrich, St. Louis, MA, USA) and sequentially incubated with primary and secondary antibodies for 1 h each in RT. Finally, the samples were stained with DAPI and mounted using a mounting medium (Brüsseler Straße, Köln, Germany). The fluorescence images were acquired via confocal microscopy (Olympus, Shinjuku, Tokyo, Japan) and analyzed using the ImageJ 4.0 software (National. Institutes of Health, Bethesda, MD, USA).

### 4.11. Western-Blot Analysis

Cells were lysed using RIPA buffer (Bio Solution, Seoul, Republic of Korea) containing a protease inhibitor cocktail and phosphatase-inhibitor cocktails 2 and 3 (Sigma-Aldrich, St. Louis, MA, USA). The lysates (30 μg total protein per sample) were subjected to 10% SDS-polyacrylamide gel electrophoresis, and the resolved proteins were transferred onto a polyvinylidene difluoride membrane (Millipore, Burlington, MA, USA). Afterward, the membrane was blocked with 1% skim milk and sequentially incubated with primary and secondary antibodies in 0.5% skim milk, and ECL Plus western-blotting detection reagent (Amersham Bioscience, Buckinghamshire, UK) was used to develop the signals. β-actin was used as a loading control. Images were obtained using the Bio-Rad ChemiDoc XRS System (Hercules, CA, USA) and analyzed using the Quantity One imaging software (Bio-Rad, Hercules, CA, USA).

### 4.12. Transfection

HepG2 cells were seeded in 24 well plates and transfected with 50 or 100 nM hsa-miR−34a mimic (Applied Biological Materials, VN, Canada) by using Lipofectamine 2000 (Invitrogen, MA, USA) according to the manufacturer’s protocol. In brief, the miR−34a mimic was mixed with 100 μL of Lipofectamine 2000 dissolved in the culture medium and split per well. After 5 h of incubation, the medium of the culture was replaced with a fresh medium without Lipofectamine 2000, and the cells were incubated overnight before being used in experiments.

### 4.13. Statistical Analysis

All the data are expressed as the mean ± SEM from three independent experiments. Statistical analysis was performed using one-way ANOVA with Tukey’s multiple comparison analysis. A *p*-value < 0.05 was considered to indicate statistical significance. The experiments were performed at least in triplicate (n = 3–6).

## 5. Conclusions

In conclusion, our investigation revealed that RAD51 was a key factor among the DNA-repair–related genes modulated by quercetin or isorhamnetin. In addition, miR−34a was significantly downregulated by B[a]P, but this effect was suppressed by quercetin or isorhamnetin co−treatment. Taken together, our investigation demonstrates that quercetin and isorhamnetin upregulate RAD51 through the downregulation of miR−34a, which can be regarded as the target against B[a]P-induced genotoxicity.

## Figures and Tables

**Table 1:** Top 15 differentially expressed genes related to DNA repair.

Gene Symbol	Annotation			
	Description	Fold Change		Normalized Read Count (log_2_)
		QB/B	IB/B	
*RAD51*	RAD51 recombinase	2.422	2.734	6.433
*ESCO2*	Establishment of sister chromatid cohesion N-acetyltransferase 2	2.351	2.691	7.394
*PARPBP*	PARP1 binding protein	2.100	2.532	6.244
*CDK1*	Cyclin-dependent kinase 1	2.061	2.736	7.082
*FANCD2*	Fanconi anemia, complementation group D2	1.975	2.622	7.983
*MND1*	Meiotic nuclear divisions 1 homolog	1.954	2.939	6.457
*BRCA2*	Breast cancer 2, early onset	1.948	2.796	6.681
*XRCC2*	X-ray repair complementing defective repair in Chinese hamster cells 2	1.915	2.405	6.988
*DTP*	Denticleless E3 ubiquitin protein ligase homolog	1.913	2.474	8.875
*POLQ*	Polymerase (DNA directed), theta	1.896	2.602	6.216
*CLSPN*	claspin	1.894	2.509	7.625
*TICRR*	TOPBP1-interacting checkpoint and replication regulator	1.854	2.213	6.882
*BRIP1*	BRCA1 interacting protein C−terminal helicase 1	1.839	2.458	7.377
*NEIL3*	Nei endonuclease VIII-like 3	1.829	2.282	6.896

## Data Availability

Not applicable.
